# Improvement of Long COVID symptoms over one year

**DOI:** 10.3389/fmed.2022.1065620

**Published:** 2023-01-09

**Authors:** Carlos R. Oliveira, Leonard A. Jason, Derya Unutmaz, Lucinda Bateman, Suzanne D. Vernon

**Affiliations:** ^1^Section of Infectious Diseases and Global Health, Department of Pediatrics, Yale University School of Medicine, New Haven, CT, United States; ^2^Section of Health Informatics, Department of Biostatistics, Yale University School of Public Health, New Haven, CT, United States; ^3^Center for Community Research, DePaul University, Chicago, IL, United States; ^4^The Jackson Laboratory for Genomic Medicine, University of Connecticut School of Medicine, Farmington, CT, United States; ^5^Bateman Horne Center, Salt Lake City, UT, United States

**Keywords:** post-acute COVID-19 syndrome, unrefreshing sleep, brain fog, Long COVID, myalgic encephalomyelitis, fatigue, post-exertional malaise

## Abstract

**Importance:**

Early and accurate diagnosis and treatment of Long COVID, clinically known as post-acute sequelae of COVID-19 (PASC), may mitigate progression to chronic diseases such as myalgic encephalomyelitis/chronic fatigue syndrome (ME/CFS). Our objective was to determine the utility of the DePaul Symptom Questionnaire (DSQ) to assess the frequency and severity of common symptoms of ME/CFS, to diagnose and monitor symptoms in patients with PASC.

**Methods:**

This prospective, observational cohort study enrolled 185 people that included 34 patients with PASC that had positive COVID-19 test and persistent symptoms of >3 months and 151 patients diagnosed with ME/CFS. PASC patients were followed over 1 year and responded to the DSQ at baseline and 12 months. ME/CFS patients responded to the DSQ at baseline and 1 year later. Changes in symptoms over time were analyzed using a fixed-effects model to compute difference-in-differences estimates between baseline and 1-year follow-up assessments.

**Participants:**

Patients were defined as having PASC if they had a previous positive COVID-19 test, were experiencing symptoms of fatigue, post-exertional malaise, or other unwellness for at least 3 months, were not hospitalized for COVID-19, had no documented major medical or psychiatric diseases prior to COVID-19, and had no other active and untreated disease processes that could explain their symptoms. PASC patients were recruited in 2021. ME/CFS patients were recruited in 2017.

**Results:**

At baseline, patients with PASC had similar symptom severity and frequency as patients with ME/CFS and satisfied ME/CFS diagnostic criteria. ME/CFS patients experienced significantly more severe unrefreshing sleep and flu-like symptoms. Five symptoms improved significantly over the course of 1 year for PASC patients including fatigue, post-exertional malaise, brain fog, irritable bowel symptoms and feeling unsteady. In contrast, there were no significant symptom improvements for ME/CFS patients.

**Conclusion and relevance:**

There were considerable similarities between patients with PASC and ME/CFS at baseline. However, symptoms improved for PASC patients over the course of a year but not for ME/CFS patients. PASC patients with significant symptom improvement no longer met ME/CFS clinical diagnostic criteria. These findings indicate that the DSQ can be used to reliably assess and monitor PASC symptoms.

## Introduction

The number of confirmed cases of COVID-19 worldwide now exceeds 624 million (1). As with previous infectious outbreaks, COVID-19 has caused significant, unresolved adverse health outcomes and disability. Current estimates of post-acute sequelae of COVID-19 (PASC), commonly referred to as Long COVID, are 49% at 4 months from acute COVID-19 infection (2).

Unexplained chronic symptoms after acute infections have been well-described for a number of other infectious diseases (3). These post-infectious syndromes are stereotypical and commonly characterized by fatigue that impairs physical function, post-exertional malaise, unrefreshing sleep, orthostatic intolerance, and cognitive impairment (4). Among the more well-established post-infectious syndromes is myalgic encephalomyelitis/chronic fatigue syndrome (ME/CFS), a condition with many symptom similarities as PASC (5, 6).

Critical to improving long-term outcomes of PASC is the identification of clinical tools that can facilitate early diagnosis, monitor trends of disability, and track progress toward recovery. Because of the symptom overlap between PASC and ME/CFS, a tool of particular interest is the DePaul Symptom Questionnaire (DSQ). The DSQ is a validated self-report questionnaire of 54 symptoms with excellent discriminant validity to diagnose ME/CFS, and high test-retest reliability to track symptoms over time (7). To assess the potential utility of the DSQ as a tool to diagnose and track recovery of patients with PASC the aims of this study were to (1) measure the prevalence and trends of DSQ-SF symptoms in a cohort of patients with PASC, and (2) assess the commonalities and differences in symptomatology and recovery between patients with PASC and ME/CFS.

## Materials and methods

### Study design and participants

For this prospective, observational cohort study, we recruited patients between the ages of 18–65 who met PASC criteria at study entry and were eligible to participate. Patients were defined as having PASC if they had documentation of previous COVID-19 (i.e., positive SARS-CoV-2 by PCR or antigen or IgG prior to receiving COVID-19 vaccination) and were experiencing symptoms of fatigue, post-exertional malaise, or other unwellness for at least 3 months that either the subject or their clinician judged to be due to COVID-19. Patients were eligible to participate if they were in good general health with no documented major medical or psychiatric diseases prior to COVID-19.

Patients were excluded from study participation if they were hospitalized for >72 h for COVID-19, had documented organ damage as a result of COVID-19 infection, or had other active and untreated disease processes that could explain their symptoms (see Supplementary Table 1 for full exclusion criteria). ME/CFS patients were recruited in 2017 (prior to the COVID-19 pandemic) and included patients between the ages of 18–65, who met the 2015 ME/CFS clinical diagnostic criteria (Supplementary Table 1) and were eligible to participate. The protocol was approved by The Jackson Laboratory Institutional Review Board study number 17-JGM-13 and the study was carried out according to United States federal regulations for the protection of human subjects as codified in 45 CFR 46. This study was carried out with adequate understanding and written consent of the participants.

### Measures

The DSQ was administered using REDCap at the start of the study and again after a 1-year follow-up (8). Using the DSQ, participants rated each symptom over the previous 6 months on a 5-point Likert-type scale in terms of frequency (e.g., 0 = None of the time, 1 = A little of the time, 2 = About half the time, 3 = Most of the time, and 4 = All of the time), and severity (e.g., 0 = Absent, 1 = Mild, 2 = Moderate, 3 = Severe, and 4 = Very severe).

### Statistical analysis

Descriptive analyses were first performed to characterize the sociodemographic characteristics of each cohort. Race/ethnicity was dichotomized to indicate whether a patient self-identified as non-Hispanic White vs. other racial and ethnic groups. The highest grade of education attained was similarly dichotomized to indicate if a participant had completed at least some post-secondary education. Fisher’s exact or Wilcoxon tests were used to assess between-group differences in categorical and continuous variables, respectively.

For the primary analysis, the percent prevalence of each symptom reported on the DSQ was estimated as the percent of patients who have values ≥2 on both the frequency and severity questions for the given symptom. To compare the difference in the prevalence of symptoms between patients with ME/CFS vs. PASC, Poisson regression was used to generate risk ratios (RR) with robust variances. A composite score was also computed for each symptom reported on the DSQ-Short Form (DSQ-SF), a subset of 14 of 54 questions from the DSQ. The composite score considered the responses from the baseline and follow-up surveys separately and was estimated by taking the mean of the frequency and severity scores for each symptom and linearly transforming it into a 100-point scale by multiplying it by 25, as previously described (9). To compare the differences in mean composite scores between PASC and ME/CFS, ordinary least squares estimation methods were used to calculate absolute risk difference (RD) with 95% CIs. To evaluate whether composite DSQ-SF scores changed over time differentially between ME/CFS and PASC, we used a fixed-effects model to compute difference-in-differences estimates between baseline and 1-year follow-up assessments. As a sensitivity analysis, we used a machine learning algorithm to examine whether a different subset of symptoms from DSQ-SF could be used for the prediction of PASC. To this end, we re-fit predictive models and applied an adaptive LASSO approach for feature selection, choosing tuning parameters and penalization with 10-fold cross-validation. Two-sided *p*-values of less than 0.05 were considered statistically significant for all comparisons. Analyses were conducted in Stata version 17.0 (StataCorp, College Station, TX, USA).

## Results

Approximately 150 people were screened for PASC, of which, 34 (22.7%) were eligible and provided informed consent to participate. Twenty-two were patients receiving clinical care for PASC at the Bateman Horne Center in Salt Lake City, Utah, and 12 were recruited from other practices for participation in the study. Of the 250 people screened for the ME/CFS cohort, 151 (60.4%) were eligible to participate and provided informed consent. Table 1 presents the demographic information for the two samples. The cohorts were comparable in terms of age, with a median of 42 years for ME/CFS and 44 years for PASC. Both groups were predominantly female, with an average of 75.7% being female. There were no significant differences in their body mass index, which averaged 25.7. There was an expected difference for time since diagnosis, as the majority of PASC patients had been diagnosed in the past year. At the time of participation in the study, 74 (49%) patients with ME/CFS had been sick for less than 4 years, and 77 (51%) patients with ME/CFS had been sick for >4 years.

**TABLE 1:** Baseline demographic characteristics of patients with ME/CFS and Long COVID.

	Total (*N* = 185)	ME/CFS (*N* = 151)	PASC (*N* = 34)	*P*-value^3^
Age, years	42 (33–52)	42 (31–54)	44 (36–49)	0.68
Sex				0.15
Female	140/185 (75.7%)	111/151 (73.5%)	29/34 (85.3%)	
Male	45/185 (24.3%)	40/151 (26.5%)	5/34 (14/7%)	
Race/ethnicity				
Non-Hispanic white	179/185 (96.8%)	148/151 (98.0%)	31/34 (91.2%)	0.093
Other race/ethnicity^1^	4/185 (2.2%)	2/151 (1/3%)	2/34 (5.9%)	
Highest education				
Secondary education or less	55/185 (29.7%)	48/151 (31/8%)	7/34 (20/6%)	0.091
Post-secondary education^2^	113/185 (61.1%)	86/151 (57.0%)	27/34 (79.4%)	
BMI	25.7 (22.5–29.8)	25.5 (21.8–30.0)	26.1 (24.4–29.0)	0.29
Time since diagnosis				<0.001
3–6 months	9/185 (4.9%)	0/151 (0.0%)	9/34 (26.5%)	
6 months to 1 year	28/185 (15.1%)	9/151 (6.0%)	19/34 (55.9%)	
1–2 years	27/185 (14.6%)	22/151 (13.9%)	6/34 (17.6%)	
2–3 years	26/185 (14.1%)	26/151 (17.2%)	0/34 (0.0%)	
3–4 years	18/185 (9.7%)	18/151 (11.9%)	0/34 (0.0%)	
>4 years	77/185 (41/6%)	77/151 (51.0%)	0/34 (0.0%)	

^1^Other race/ethnicity: Asian (*n* = 2), Latino (*n* = 2).

^2^Post-secondary education: University (*n* = 104); Vocational school (*n* = 9).

^3^Wilcoxon tests were used to compare between-group differences in age and body mass index (BMI).

All other variables were compared using Fisher’s exact test.

**FIGURE 1:**
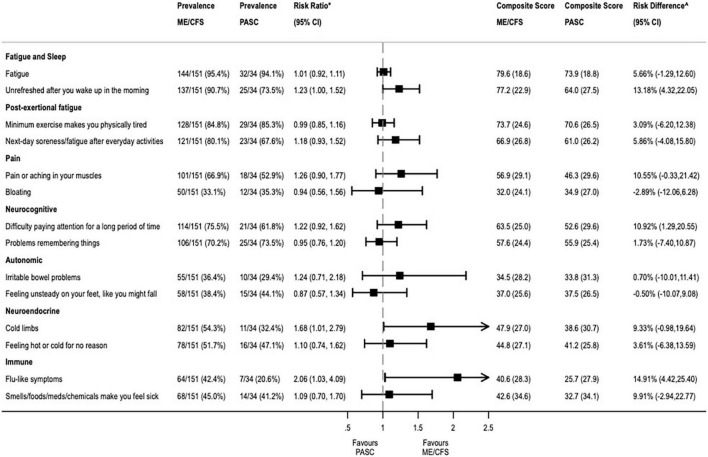
Differences in mean composite score of baseline symptoms between PASC and ME/CFS.

Among the PASC patients, the most prevalent complaints included general fatigue (*n* = 32, 94%), sleep problems (*n* = 25, 74%), myalgias (*n* = 18, 53%), and neurocognitive symptoms, like problems remembering things (*n* = 25, 74%) or difficulties paying attention for a long period of time (*n* = 21, 62%). Figure 1 provides the mean composite score and percent prevalence of each symptom at baseline for the two samples. There was a significant difference in the baseline mean composite score for two symptoms (“Unrefreshed in the morning” and “Flu-like symptoms”), with those in the ME/CFS group being more likely than those with PASC to report unrefreshing sleep (RD 13.2%, 95% CI: 4.3–22.1%), and more likely to report flu-like symptoms (RD 14.9%, 95% CI: 4.4–25.4%). In the sensitivity analysis, the same 2 out of the 14 composite DSQ-SF symptoms, “Unrefreshed in the morning” and “Flu-like symptoms,” were selected as predictive of ME/CFS using adaptive LASSO regression (Table 2). These variables were the same identified in our primary analysis, confirming the importance of these predictors in our sample.

**TABLE 2:** Results of LASSO regression with adaptive cross-validation.

Description	Lambda	No. of non-zero coef.	CV mean error
First lambda	0.1689859	0	1.076982
First* variable added	0.1539737	1	1.074633
Second** variable added	0.0381405	2	1.042904
Lambda before selected	0.0021324	2	1.031949
Selected lambda^∧^	0.0019429	2	1.031949
Lambda after selected	0.0017703	2	1.031949
Last lambda	0.0005282	2	1.031979

^∧^lambda selected by cross-validation (CV) in final adaptive step.

*First variable = feeling flu-like symptoms.

**Second variable, feeling unrefreshed after you wake up in the morning.

Follow-up surveys were completed by 67% (124/185) of participants (PASC: 20/34; ME/CFS: 104/151). Differences in the percent prevalence and mean composite symptom scores at follow-up between PASC and ME/CFS are shown in Supplementary Figure 1. Mean composite symptom scores did not significantly change over time in patients with ME/CFS. However, PASC patients showed a significantly greater reduction in symptoms across 5 out of the 14 DSQ-SF items over time (Table 3).

**TABLE 3:** Differences in mean composite scores between baseline and 1-year follow-up.

	Mean (SD) composite score^∧^						Diff-in-Diff (95% CI)	*P*-value
	ME/CFS (*N* = 104)			PASC (*N* = 20)				
	Baseline	1-year	*P*-value	Baseline	1-year	*P*-value		
**Fatigue and sleep**								
Fatigue	80.3 (18.4)	74.9 (21.5)	0.053	70.6 (19.1)	56.2 (23.1)	**0.039**	−9.09 (−17.30, −0.88)	**0.03**
Unrefreshed after you wake up in the morning	76.8 (22.7)	70.4 (25.5)	0.057	59.4 (29.5)	45.0 (24.1)	0.100	−7.83 (−19.51, 3.86)	0.189
**Post-exertional fatigue**								
Minimum exercise makes you physically tired	74.3 (25.2)	70.8 (25.3)	0.32	66.9 (30.7)	51.9 (32.0)	0.14	−11.48 (−21.32, −1.64)	**0.022**
Next-day soreness/fatigue after everyday activities	67.7 (27.6)	66.4 (24.4)	0.72	56.2 (26.7)	46.9 (31.1)	0.31	−8.06 (−19.86, 3.74)	0.181
**Pain**								
Pain or aching in your muscles	56.4 (29.8)	54.4 (25.3)	0.6	40.0 (29.7)	30.6 (29.1)	0.32	−7.69 (−17.04, 1.65)	0.107
Bloating	28.6 (22.4)	30.6 (23.5)	0.54	31.2 (27.7)	25.6 (26.7)	0.52	−7.42 (−17.00, 2.16)	0.129
**Neurocognitive**								
Difficulty paying attention for a long period of time	63.8 (25.4)	61.7 (24.0)	0.53	47.5 (29.4)	37.5 (26.9)	0.27	−7.9 (−17.49, 1.69)	0.106
Problems remembering things	58.1 (23.7)	56.8 (23.0)	0.7	53.8 (23.0)	38.1 (22.4)	**0.036**	−14.22 (−22.55, −5.89)	**0.001**
**Autonomic/neuroendocrine**								
Irritable bowel problems	32.3 (28.0)	34.7 (28.3)	0.54	32.5 (31.5)	23.1 (25.1)	0.30	−11.54 (−22.41, −0.67)	**0.041**
Feeling unsteady on your feet, like you might fall	36.3 (24.6)	35.0 (23.8)	0.69	42.5 (28.8)	23.8 (22.5)	0.027	−17.34 (−27.79, −6.89)	**0.02**
Cold limbs	45.8 (26.0)	39.7 (26.7)	0.097	38.8 (30.6)	29.4 (23.4)	0.28	−3.36 (−15.41, 8.70)	0.585
Feeling hot or cold for no reason	46.4 (26.1)	44.8 (26.7)	0.66	38.8 (24.0)	31.9 (24.5)	0.38	−5.28 (−16.76, 6.20)	0.367
**Immune**								
Flu-like symptoms	39.7 (27.0)	35.0 (26.3)	0.2	21.9 (23.9)	11.9 (18.8)	0.15	−5.27 (−15.19, 4.65)	0.297
Some smells/foods/meds/chemicals make you feel sick	43.5 (35.0)	44.1 (30.7)	0.91	30.0 (34.0)	26.2 (29.5)	0.71	−4.01 (−16.77, 8.74)	0.538

^∧^Composite score: Estimated as the mean of the frequency and severity scores for each symptom linearly transformed to a 0–100, presented as mean (SD).

Diff-in-Diff: Difference-in-difference; estimated as the change in the pooled composite symptom score over time of the PASC group minus that of the ME/CFS group.

Bold *p*-values indicate statistical significance.

## Discussion

In this ongoing prospective cohort of patients, we build on the growing body of evidence describing the commonalities in symptomatology between patients with PASC and those with ME/CFS (6, 10, 11). We found that PASC patients had high rates of ME/CFS symptoms, including post-exertional malaise, cognitive impairment, and sleep disruptions. Notably, nearly all patients with PASC met criteria for ME/CFS using the Institute of Medicine clinical diagnostic criteria for ME/CFS (12), and only minor differences in the relative frequency of two symptoms were detected between PASC and ME/CFS cohorts. Given that the prevalence of ME/CFS in the general population is less than 1% (13), these data would suggest that this previously validated ME/CFS questionnaire could also be leveraged for the detection and diagnosis of PASC. By reducing criterion variance, the largest source of diagnostic unreliability for case definitions, the DSQ-SF may aid in the critical task of selecting those with the illness and increasing diagnostic sensitivity.

We build on the recent work of Jason et al. (14) who utilized the DSQ to compare a sample of people with ME/CFS to those who had not recovered from COVID in two significant respects. First, we focused on the analysis of the DSQ-SF, which includes just 14 of the original 54 symptoms queried in the full DSQ. We did this in part due to its brevity and high acceptability within the medical community. Because it can be completed in approximately 5 min, it can easily be implemented in a routine clinical setting for screening purposes (15, 16). The DSQ-SF has been translated into many languages and is being used as an outcome measure in a PASC clinical trial in Ireland (17).

All subjects in this study answered the full DSQ at each time point. We chose to analyze the 14 questions that make up the DSQ-SF because of its favorable psychometric qualities and to determine whether these 14 items could be used as a screening tool in larger PASC studies such as RECOVER. Independent investigators have confirmed the reliability and validity of the individual items in the survey. As an example, Murdock et al. found no problems of ceiling effects with DSQ, which were evident with two other patient-reported symptom measures (18). Items and scales on the DSQ have also been related to biological findings among patients with ME/CFS, including vision-related abnormalities (19), QEEG brain recordings (20), and autonomic dysfunction (21).

Second, this study builds on previous work by deploying the standardized DSQ-SF longitudinally in a cohort of PASC patients. Reliable instruments are needed not only for diagnosis but also for monitoring patients with PASC. The DSQ-SF could be used as a brief assessment to quantify and trend the intensity of symptoms over time in a standardized manner. In our study, while the similarities between PASC and ME/CFS at baseline were considerable, we found a substantial reduction in self-reported symptoms in PASC patients at 1-year compared to the pre-pandemic ME/CFS group. This suggests that symptoms of PASC may slowly resolve over time in many subjects.

We recognize that this study has some potential limitations. We used a convenience sample that was limited to adults who sought care at specialty clinics. Thus, findings may not be generalizable to other populations, particularly in children who may have different PASC experiences. As the DSQ-SF was developed for ME/CFS, there will be symptoms that it will not detect, such as persistent anosmia or hair loss, which are rarely experienced by patients with ME/CFS but are common in PASC. Future studies will be needed to determine how well this tool can discriminate between PASC and other chronic conditions. Despite these limitations, these data provide evidence that supports the use of DSQ-SF as a framework for screening, assessing, and monitoring PASC symptoms.

## Data availability statement

The raw data supporting the conclusions of this article will be made available by the authors, without undue reservation.

## Ethics statement

The studies involving human participants were reviewed and approved by the Jackson Laboratory Institutional Review Board. The patients/participants provided their written informed consent to participate in this study.

## Author contributions

CRO, DU, LB, and SDV had full access to all the data in the study and took responsibility for the integrity of the data and the accuracy of the data analysis. CRO, LJ, and SDV drafted the manuscript. CRO contributed to the statistical analysis. DU obtained the funding. SDV contributed to the administrative, technical, or material support. All authors contributed to the concept and design, acquisition, analysis, or interpretation of data, drafting of the manuscript, and critical revision of the manuscript for important intellectual content.
