# Supplementary material for: Improvement of Long COVID symptoms over one year
Source: Front Med (Lausanne). 2023 Jan 9;9:1065620. doi: 10.3389/fmed.2022.1065620 (PMC9868805; doi:10.3389/fmed.2022.1065620)

**Supplemental Material**

**Supplemental Table 1. Study definitions and exclusion criteria**

| **ME/CFS criteria (2015 IOM report)** | - A substantial reduction or impairment in the ability to engage in pre-illness levels of occupational, educational, social, or personal activities that persists for more than 6 months - Fatigue, which is often profound, is of new or definite onset (not lifelong), is not the result of ongoing excessive exertion, and is not substantially alleviated by rest - Post-exertional malaise - Unrefreshing sleep, plus either cognitive impairment or orthostatic intolerance. - Symptoms are present at least half of the time and are moderate to severe |
| --- | --- |
| **PASC criteria (WHO)** | - Had documentation of a previous SARS-CoV-2 infection   - Positive SARS-CoV-2 by PCR or antigen   - Positive IgG prior to receiving COVID-19 vaccination - Experiencing symptoms of fatigue, exercise intolerance, or other unwellness for at least 3 months that either the subject or their clinician judged to be due to COVID-19 - No other condition that could explain the symptoms |
| **Exclusion criteria** | - Had severe COVID-19:   - Was hospitalized for >72 hours during acute infection   - Had documented organ damage as a result of COVID-19 - Has comorbidity that could explain PASC symptoms   - Addison’s disease, Cushing’s Syndrome, hypothyroidism, hyperthyroidism, anemia, iron overload syndrome, diabetes mellitus, cancer, untreated primary sleep disorders (e.g., upper airway resistance syndrome or obstructive or central sleep apnea), rheumatological disorders (e.g., rheumatoid arthritis, lupus, polymyositis and polymyalgia rheumatica), immune disorders, neurological disorders (e.g., multiple sclerosis, Parkinsonism, myasthenia gravis and untreated B12 deficiency), active infectious diseases (e.g., tuberculosis, chronic hepatitis, and acute Lyme disease). - Has psychiatric disorders that could alter the perception of reality or ability to communicate clearly or impair physical health and function (e.g., bipolar with psychotic features, active anorexia or bulimia, major melancholic depression, and active substance abuse). |

**Supplemental Figure 1. Differences in the percent prevalence and mean composite symptom scores between PASC and ME/CFS at 12 month follow-up**


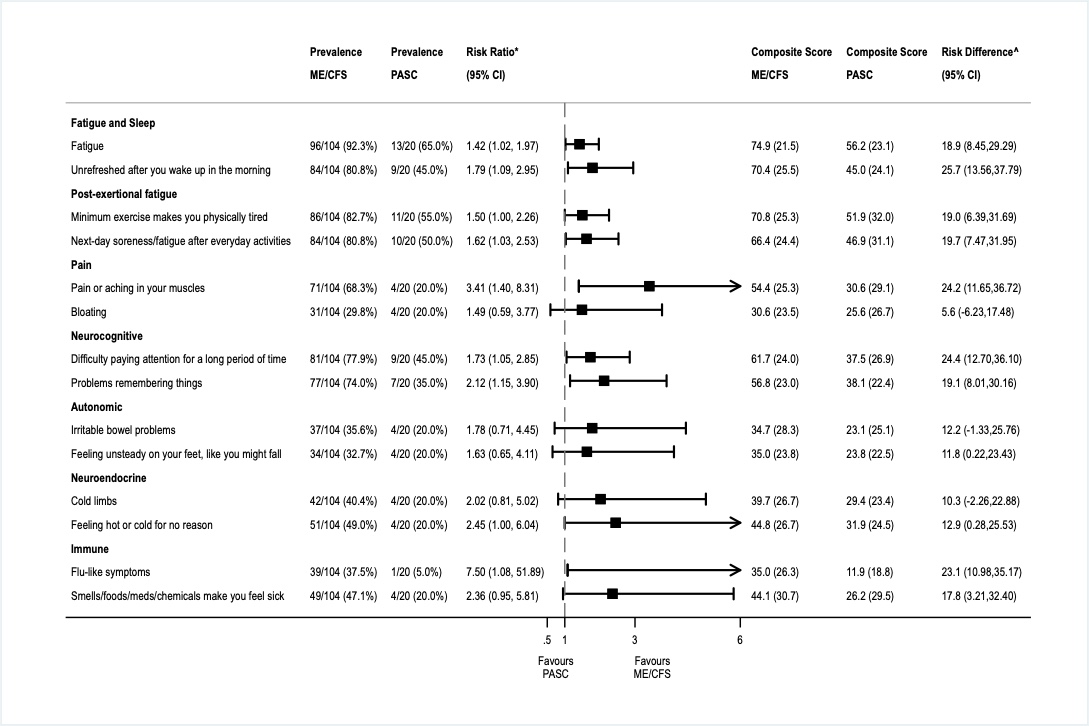

Supplement: Supplementary file 1 [file Data_Sheet_1.docx]
